# 

**Published:** 1805-02-01

**Authors:** 


### To CORRESPONDENTS.

Dr. Hall's paper on Catarrh, Mr. Hume's paper on modern Pharmacopœias, and a Review of Dr. Jackson's "Remarks on the Constitution of the Medical Department of the British Army, with a Detail of Hospital Management," will appear in our next Number.

